# Online information for patients on cancer diets – a comparison of German- and English-language websites

**DOI:** 10.1186/s12911-026-03529-7

**Published:** 2026-04-30

**Authors:** Juliane Hauff, Lena Josfeld, Jutta Hübner

**Affiliations:** 1 KIM II, University Hospital Jena, Am Klinikum 1, 07747 Jena, Germany; 2 Thüringische Krebsgesellschaft e.V., Jena, Germany

**Keywords:** Cancer diets, Patient information, Internet, Web-based information

## Abstract

**Background:**

The internet has become an important source of information for cancer patients. Numerous websites provide nutritional advice that promises benefits for the outcome of cancer therapy. The aim of our study was to evaluate and compare the online information about cancer diets on German- and English-language websites.

**Methods:**

A patient’s online search was simulated using the search engines Google and Bing. Websites were evaluated by means of content and formal criteria according to a standardized instrument.

**Results:**

The analysis of 31 websites revealed heterogeneous quality regarding content and formality, distributed evenly among the German- and English-language websites. The quality of content and formality does not correlate with the website’s order of appearance in a browser-based search.

**Conclusions:**

The high discrepancy in quality of content and formality represents a risk for cancer patients, who are searching for information online. Content of poor quality and formality increases the risk of mal-information and consecutive false decisions on diet. It results in the decline of therapy response, an increased probability of therapeutic toxicity and a poorer prognosis in general. The visibility of high-quality websites needs to be improved.

## Background

When being diagnosed with cancer, it is a natural step for patients and family members to look for information about the disease, prognosis and treatment options. Next to cancer-specific information such as stage of disease and specific diagnosis information, patients often seek knowledge of available treatments, treatment options and side effects of treatment [1–4]. The gain of information helps patients to better cope with the disease, to reduce anxiety, and it enables a more involved decision-making, resulting in a patient-centered doctor-patient-relationship and a greater contentment with treatment choices [5, 6].

Although health care providers remain an important source of information, there has been a rapid increase in the number of people searching for health recommendations on the internet. While the older generation relies mainly on the information provided by their physician, younger generations require additional and complex facts from a broader range of sources [7, 8]. In 2005, approximately 61% of cancer patients were estimated to search for health information on their type of cancer online [3, 9]. In the following decade the prevalence of internet consultation after a cancer diagnosis increased to 89% in 2017 [9] and can be expected to rise even more within the next years.

For patients, who feel that the information offered to them by their health care provider is insufficient, or who simply want to know as much as possible about their disease, the internet provides plenty advantages [10, 11]. Besides the risks of feeling overwhelmed and misunderstanding and misinterpreting facts, searching online is a convenient and easy way to anonymously access an inexhaustible volume of information [8].

Along with conventional treatment options, patients also search for alternative therapies, which convey a feeling of control over the therapies, and which offer hope for times when conventional treatments fail [1, 4]. Most recommendations for complementary and alternative medicine (CAM) for cancer patients include dietary approaches. The belief that cancer can be cured solely by nutritional therapies resulted in the emergence of various cancer diets, which differ tremendously from one another [12]. The alkaline diet for example is based on a nutrition rich in vegetables, low-sugar fruits, nuts and legumes with the avoidance of sugar, grains, dairy and meat. The Gerson’s regimen demands a raw vegetable diet with fruit and vegetable juices, salt and protein restriction, a potassium and iodine administration as well as coffee enemas up to four times a day. The Breuss diet on the other hand allows only tea and vegetable juices for a period of at least 42 days, with the aim to starve the tumor during this fasting [12, 13]. While a healthy nutrition is important for patients and plays a role in the prevention of cancer, there is little to no evidence from clinical studies that a cancer diet alone can cure cancer [13, 14]. Besides patients who regard cancer diets as an option for healing cancer, many patients look for information on healthy nutrition with the aim of enhancing well-being and quality of life during and after the treatment, boosting the immune system, and reducing side effects of cancer treatment [13, 15–18]. Relying on cancer diets as a cure may delay cancer treatment, resulting in the increase of cancer-related symptoms or at worst in a progress or relapse of disease [13]. Moreover, restrictive diets during and after cancer treatment may lead to malnutrition. Since malnutrition already affects 40% of hospitalized cancer patients, resulting in the decline of therapy response, an increased probability of therapeutic toxicity and a poorer prognosis in general, it is crucial for physicians to educate their patients about the benefits, side effects and dangers of complementary and alternative approaches such as cancer diets [19].

As many patients report not receiving enough information on nutrition from the oncologist, the internet is a frequently used source of information [20]. While patients most probably prefer the national, German-language websites, many people today also are familiar with information in English language. Accordingly, we decided to analyze the quality of web-based patient information on nutrition in cancer in German and in English language, with respect to content as well as format according to national and international criteria for written patient information. The purpose of our study was to assess, whether lay-people looking for such information using a search engine find evidence-based well-presented information by a simple search.

## Methods

**Fig. 1:**
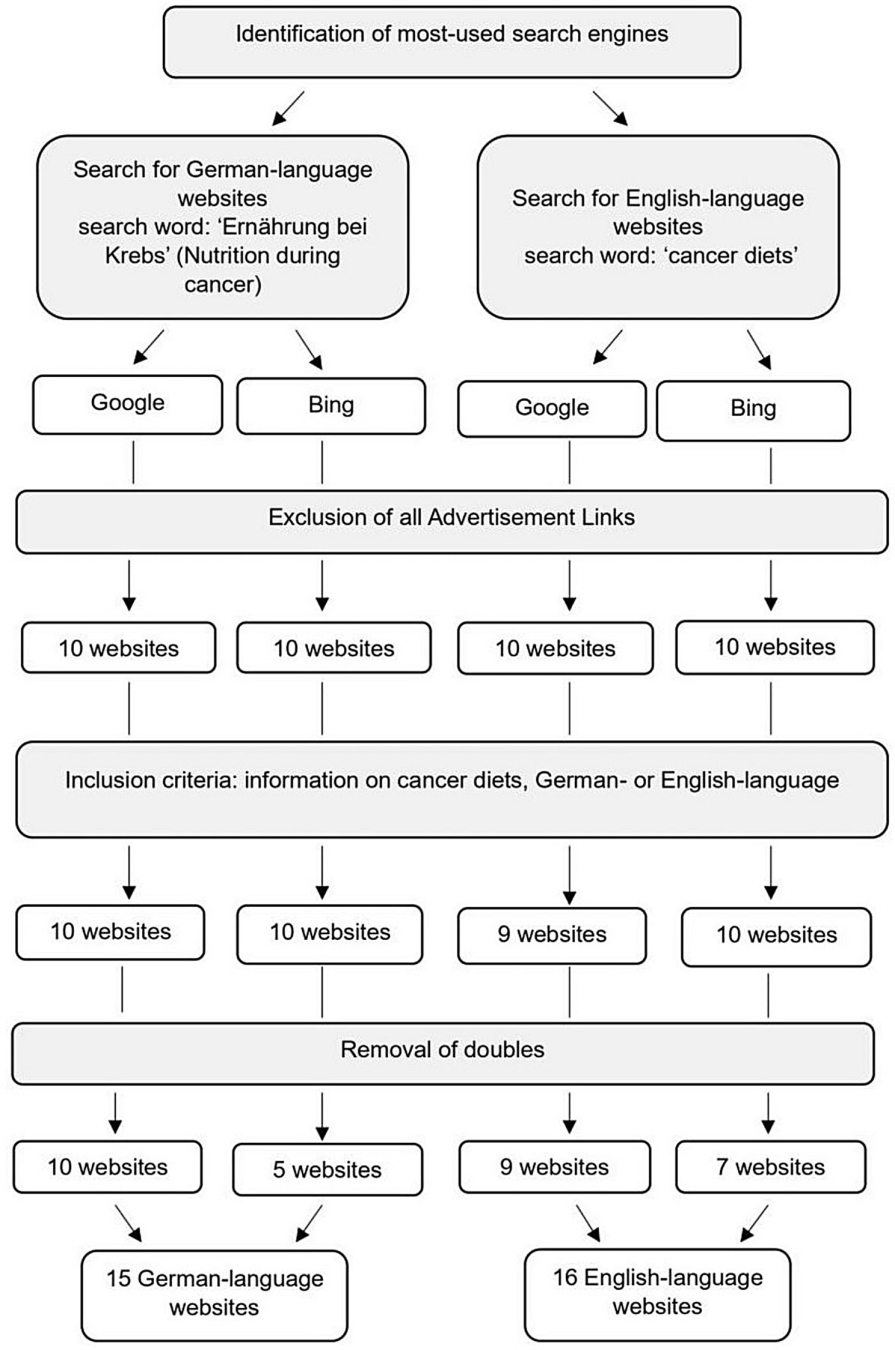
Process of website selection

In September 2023, we started by identifying the two most used search engines in German- and English-speaking countries [21, 22]. Figure 1 depicts our process of the website selection. We simulated a patient’s search for information using the search engines Google and Bing for both, the German and English search. For the purpose of avoiding any personalization problems due to our location and previous searches, we cleared our web browser’s cache and cookies and turned off the location tracking ahead of the search. In order to obtain equivalent content, language adapted search words used were ‘Ernährung bei Krebs’ (Nutrition during cancer) and ‘cancer diets’ which we identified in our former study as the search terms most often used by lay people [23, 24]. Analogous to the previous study by Herth et al. [23] and comparable to the search strategy of a lay person, we typed in the search terms into the search bar of both search engines, Google and Bing.

The inclusion criteria for our study contained the presence of information on cancer diets, the websites being in German- or English-language and the absence of advertisement. After excluding all Advertisement Links (Google Ads) we considered the first ten websites of each search engine and each language for further assessment, as most people do not look at websites that appear past the first page [25, 26]. One English-language website did not meet our inclusion criterion of containing any information about cancer diets and was therefore excluded. Eight Websites appeared on Bing and Google (five German-language, three English-language) but were only assessed once, resulting in a total of fifteen German-language and sixteen English-language websites.

Next, we assessed the selected websites focusing on content and format, using an adapted version of a catalogue of criteria established in a previous study by Liebl et al. [24] as shown in Table 1. This catalogue is based on criteria for written patient information given on the EbM Network (German Network for Evidence-based Medicine) and the ÄZQ (Agency for Quality in Medicine) [27] as well as certification criteria and instruments for website evaluation by patients. This includes certificates like the HONcode, that validates the trustworthiness of websites with medical information, based on a catalogue of formal criteria [28], Discern, an online instrument that helps users of patient information to assess the quality of presented information [29] and the ten afgis criteria for transparency that were developed to evaluate the trustworthiness and reliability of online patient information [30].

**Table 1:** Criteria for the evaluation of websites

**Content criteria**
Completeness
Expertise
Explication of objectives and target audience
Fair balance/neutrality
Precision
Relevance
Appropriate, intelligible figures
Suitability to support shared decision making, scientific evidence and timeliness
No statements on topics without evidence
Information on additional resources and references
Focus on the patient
Layout aspects
Quality management
Clear arrangement of information
Labeling of missing evidence and risks
**Formal criteria**
Transparency concerning provider, supporter
Transparency concerning funding, advertisement
Privacy protection
Completeness of information on sources of evidence
Language adapted to the needs of the target group
Possibilities of feedback and participation for users

The websites were individually evaluated according to the content and formal criteria by two of the authors (JH, JHü) from September to October 2023. We used a 3-point Likert Scale, a score of 1 equaling full accordance with the criterion, 2 being given for partial accordance with some drawbacks and 3 standing for violation of the criterion or deviation in critical points. Every criterion received an individual score according to our Likert Scale by each rater. Kendall’s coefficient of concordance (Kendall’s W) was used to assess the inter-rater agreement.

The evaluation scores of all criteria of both authors were added for each website. For an easier and better comparison of the different categories we normalized the total score of each website to a range of 0-100 by transforming the highest score into a score of 100 and putting all other scores in relation. Additionally, we ranked all websites according to their normalized individual content, formal and overall score and compared the number of German-language websites with the number of English-language websites that ranked in the best rated third, as well as the middle and lowest rated third.

## Results

Overall, 31 websites were evaluated by two of the authors (JH, JHü) in regard to the content and formal criteria shown in Table 1. Overall concordance between the two raters was good at W = 0.836 (*p* = 0.01). Concordance for the English-language websites alone was even higher (W = 0.911, *p* = 0.03). For the German-language websites, concordance among raters failed to reach significance (W = 0.752, *p* = 0.10), so the null hypothesis (agreement between raters is due to chance) cannot be rejected.

Tables 2 and 3 show the evaluated German- and English-language websites and their order of appearance on Google and Bing. We created the depicted list of websites by naming the websites from Google at first, followed by the remaining websites from Bing. Each site received an individual score per rater for content criteria, as well as for formal criteria, which were ultimately summed up to form an overall evaluation score. The total for content criteria ranged from 43 to 89 points, a lower score standing for a higher quality of information concerning completeness, expertise, relevance etc. The range for formal criteria was 19 to 34 points, a lower score functioning as an indicator for good transparency, privacy protection and consideration of the user’s needs. Finally, the overall scores varied between 65 and 122 points. As the ranges are different, all scores were additionally normalized to a scale from 0 to 100 for better comparison.

**Table 2:** Evaluated German-language websites

Website Number	German-language Websites	Numberon Google search	Number on Bing search	Overall ranking	Content ranking	Formal ranking
1	Deutsche Krebsgesellschaft (German cancer society) Link: https://www.krebsgesellschaft.de/basis-informationen-krebs-bewusst-leben-ernaehrung.html	1	6	5	5	3
2	Deutsche Krebshilfe (German cancer aid) Link https://www.krebshilfe.de/infomaterial/Blaue_Ratgeber/Ernaehrung-bei-Krebs_BlaueRatgeber_DeutscheKrebshilfe.pdf	2	5	7	9	2
3	Helios magazine (Privat Health care provider) Link: https://www.helios-gesundheit.de/kliniken/berlin-buch/unser-angebot/unsere-fachbereiche/onkologisches-zentrum-berlin-buch/die-10-effektivsten-anti-krebs-lebensmittel/	3	/	11	12	6
4	Tumorzentrum München (Tumor center Munich) Link: https://www.tumorzentrum-muenchen.de/ernaehrung.html	4	/	6	5	5
5	Krebsinformationsdienst (Cancer information service) Link: https://www.krebsinformationsdienst.de/leben/alltag/ernaehrung/index.php	5	9	1	2	1
6	NDR Ratgeber (Online Magazine of a German broadcast provider) – The right nutrition during cancer Link: https://www.ndr.de/ratgeber/gesundheit/Die-richtige-Ernaehrung-bei-Krebs,krebs394.html	6	3	12	11	14
7	Apotheken Umschau (Pharmacy magazine) Link: https://www.apotheken-umschau.de/krankheiten-symptome/krebs/essen-und-krebs-was-schuetzt-was-schadet-718201.html	7	/	4	4	6
8	Krebs (cancer) Link: https://www.krebs.de/leben-mit-krebs/ernaehrung-bei-krebs	8	2	2	1	10
9	Zentralklinik Bad Berka (Hospital Bad Berka) Link: https://www.zentralklinik.de/unsere-medizin/zertifizierte-zentren/enets/informationen/krebs-und-ernaehrung.html	9	/	10	10	13
10	Was essen bei Krebs (What to eat if you have cancer) Link: https://www.was-essen-bei-krebs.de/	10	/	3	3	3
11	Praxis Dr. Dornschneider (Dr. Dornschneider’s practice) Link: https://dr-dornschneider.de/ernaehrung-bei-krebs/#:~:text=Die%20bestm%C3%B6gliche%20Ern%C3%A4hrung%20bei%20Krebskranken%20sollte%20daher%20reich,hemmender%20Effekt%20auf%20das%20Tumorwachstum%20wird%20jedoch%20angenommen.	/	1	15	15	11
12	NDR Ratgeber (Online Magazine of a German broadcast provider) – Nutrition during cancer Link: https://www.ndr.de/ratgeber/gesundheit/Ernaehrung-bei-Krebs,krebs316.html	/	4	13	14	11
13	Gesundheit (Health) Link: https://www.gesundheit.de/krankheiten/krebs/galerie-ernaehrung	/	7	9	8	6
14	Smartessen (Smart eating) Link: https://smartessen.de/ernaehrung-bei-krebs/	/	8	14	13	15
15	Spektrum, Ernährung bei Krebs (Nutrition during cancer) Link: https://www.spektrum.de/news/ernaehrung-bei-krebs-der-zucker-darf-bleiben/1963054	/	10	8	5	9

**Table 3:** Evaluated English-language websites

Website Number	English-language Websites	Numberon Google	Numberon Bing	Overall ranking	Content ranking	Formal ranking
1	Johns Hopkins Medicine Link: https://www.hopkinsmedicine.org/health/conditions-and-diseases/cancer/cancer-diet-foods-to-add-and-avoid-during-cancer-treatment#:~:text=Plant%2Dbased%20Proteins,%2 C%20legumes%2 C%20nuts%20and%20seeds.	1	1	8	9	11
2	WebMD Link: https://www.webmd.com/cancer/cancer-diet	2	/	6	7	1
3	Stanford Medicine Health Care Link: https://stanfordhealthcare.org/medical-clinics/cancer-nutrition-services/during-cancer-treatment.html	3	/	7	4	14
4	Michigan Medicine Link: https://healthblog.uofmhealth.org/wellness-prevention/best-diets-for-cancer-patients-and-cancer-survivors	4	2	16	16	15
5	Everyday Health Link: https://www.everydayhealth.com/cancer/diet-cancer-what-you-need-know-eat-feel-your-best-while-fighting-cancer/	5	/	8	11	8
6	MD Anderson Cancer Center Link: https://www.mdanderson.org/prevention-screening/manage-your-risk/diet.html	6	8	13	14	9
7	American Cancer Society Link: https://www.cancer.org/treatment/survivorship-during-and-after-treatment/coping/nutrition.html	7	/	5	6	2
8	Cancer Support Community Link: https://www.cancersupportcommunity.org/diet-nutrition-during-cancer-treatment	8	/	3	2	7
9	Rogel Cancer Center Link: https://www.rogelcancercenter.org/living-with-cancer/nutrition/five-popular-diets-are-they-right-cancer-survivors	9	/	8	7	13
10	Cancertutor Link: https://www.cancertutor.com/cancer-diet/	/	3	14	15	11
11	American Institute for Cancer Research Link: https://www.aicr.org/cancer-prevention/food-facts/	/	4	1	1	2
12	Verywell Health Link: https://www.verywellhealth.com/cancer-diet-5094145	/	5	2	2	5
13	Eat This, Not That! Link: https://www.eatthis.com/cancer-fighting-diets/	/	6	8	10	9
14	Cascoon Link: https://cascoon.dvrdns.org/nutrition/cancer-and-diet	/	7	15	12	15
15	National Cancer Institute Link: https://www.cancer.gov/about-cancer/causes-prevention/risk/diet	/	9	4	5	5
16	Wikipedia Link: https://en.wikipedia.org/wiki/Diet_and_cancer	/	10	12	13	2

**Fig. 2:**
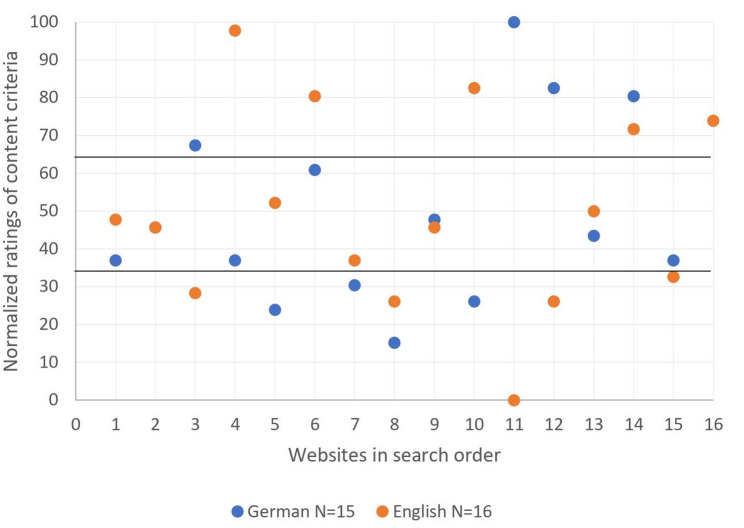
Comparison of German- and English-language websites in content criteria

**Fig. 3:**
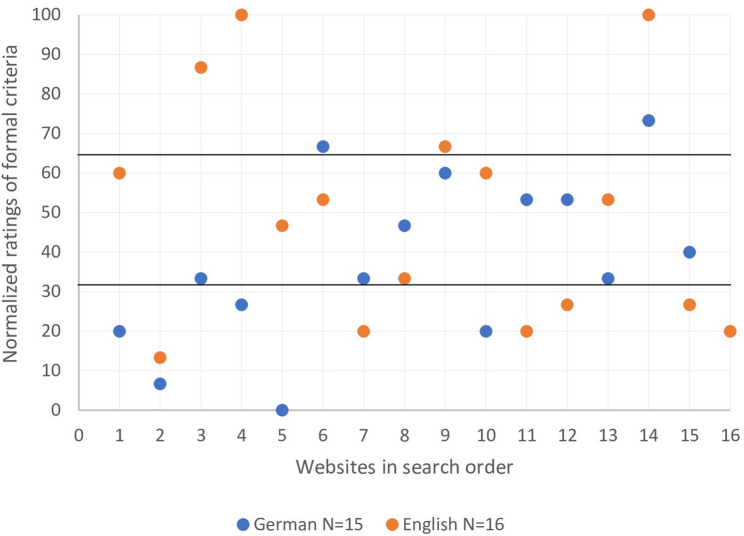
Comparison of German- and English-language websites in formal criteria

**Fig. 4:**
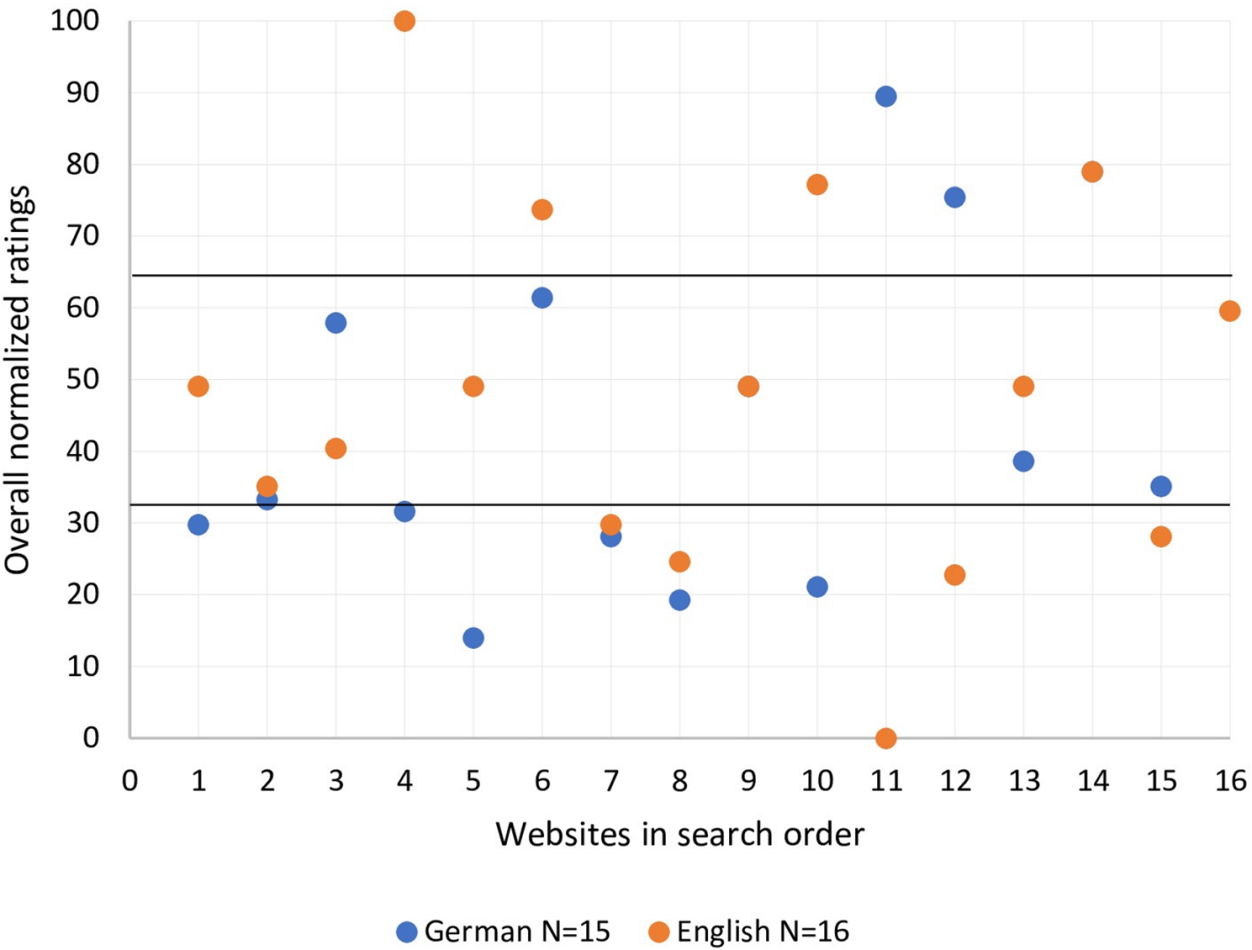
Overall ratings of German- and English-language websites

Figures 2 and 3 display the distribution of normalized content and formal ratings for the German- and English-language websites. Both figures show a similar arrangement of ratings. Overall, most websites received a medium high normalized score between 33.3 and 66.6 points. As shown in Fig. 2, the distribution of the remaining websites was even, with nine websites receiving a normalized score between 0 and 33.3 points, equaling a good content rating, and nine websites receiving a normalized score of 66.6 to 100 points, therefore having high ratings and low quality of content. Regarding the distribution of the normalized formal ratings, Fig. 3 displays a more uneven spread. Thirteen websites received a medium high normalized score between 33.3 and 66.6 points, followed by eleven websites with a score between 0 and 33.3 points, equaling a good formal rating and only seven websites with a normalized score of 66.6 to 100 points, equaling low quality of formal criteria. A detailed analysis of both figures reveals a more evenly spread of ratings for the English-language websites between the top, medium and bottom third, whereas the German rankings show a more notable divergence in the distribution of the German-language website’s ratings between the three thirds. Nonetheless, neither the differences in the distribution of German- and English-language website’s rating in Fig. 2, nor in Fig. 3 are statistically significant. Figure 4 displays the distribution of the normalized overall ratings (content + formal), demonstrating once more no significant differences between the German- and English-language websites.

Table 4 individually lists the three best websites for each section (content, formal and overall ranking). The best score for content ranking was achieved by the ‘American Institute for Cancer Research’, followed by the German websites ‘krebs.de’(‘cancer’) and ‘Krebsinformationsdienst’ (‘cancer information service’). The best formal scores were dominated by the German websites ‘Krebsinformationsdienst’ (‘cancer information service’) and ‘Deutsche Krebshilfe’ (‘German cancer aid’) followed by the American website ‘webMD’. Finally, the three best overall scores were dispersed among one American (‘American Institute for Cancer Research’) and two German websites (‘Krebsinformationsdienst’(cancer information service’), ‘krebs.de’ (‘cancer’)).

**Table 4:** Best-ranked websites

	3 best-ranked websites	Total Rating score	Normalized rating score	Origin
Content ranking	https://www.aicr.org https://www.krebs.de https://www.krebsinformationsdienst.de	43 50 54	0 15,2 23,9	USA Germany Germany
*Formal ranking*	https://www.krebsinformationsdienst.de https://www.krebshilfe.de https://www.webmd.com	19 20 21	0 6,7 13,3	Germany Germany USA
*Overall ranking*	https://www.aicr.org https://www.krebsinformationsdienst.de https://www.krebs.de	65 73 76	0 14,4 19,3	USA Germany Germany

Furthermore, our study revealed that the order in which websites appear after a Google or Bing search does not correlate with good quality of content and formality. For instance, Fig. 4; Table 3 show that in the English search the best overall website (‘American Institute for Cancer Research’) occurred as search result number 4 on Bing and was not even included in the first ten search results on Google, whereas the website with the worst overall rating (‘Healthblog Michigan Health’) appeared in 2nd place on Bing and 4th place on Google. The same circumstance can be found while looking individually at the content and formal ratings in Figs. 2 and 3, combined with Tables 2 and 3, showing that websites with the best ratings often do not appear as the first websites on a Google results page.

All in all, no significant difference in the quality of information on cancer diets between German- and English-language websites can be found. However, it becomes clear that not all websites fulfill each criterion (content or formal) to the same extent and that there is a broad-spectrum regarding quality of content and formality for both, the German- and English-language websites.

## Discussion

The goal of our study was to assess, whether lay-people, looking for online patient information on nutrition in cancer in German and in English language, find evidence-based well-presented information by a simple search, as the establishment and recommendation of health portals that link to advisable websites can be a promising option for the gathering of information.

Our analysis of German- and English-language websites reveals that there is a high discrepancy in quality and formality standards between the individual websites, that ought to inform patients about cancer diets. The findings of our study show that there are websites in both, the German- and English-language, that can be recommended to cancer patients. Yet, the visibility of these recommendable websites on the World Wide Web and the distinguishability from websites with low quality of content and formality needs to be improved.

In websites of both languages, good quality of content does not necessarily correlate with good formality, since not every website fulfills the content and formality criteria to the same extent. In their previous analysis of National Cancer Center websites, Brauer et al. also reported a high variability in content quality. Compared to our results of nine websites (29%) receiving a good rating score for content, their study shows similar results, with only 17% of websites receiving an excellent rating score [31]. Similar findings are reported in a past website analysis on nutritional information for cancer patients in the UK, revealing the containment of non-evidence-based or false information, no correlation between good quality of content and formality, and with the majority of websites not being within the acceptable readability level [32]. These findings could be caused by the presumption that high quality content is often provided by cancer organizations, who focus on giving information to experts (doctors, nutritionists etc.) and therefore miss out on adapting to a patient’s individual need regarding the level of language adaptation, transparency, or participation. Thus, it is a very difficult task to meet the needs of every website user in a written format. This could explain why the two authors (JH, JHü) failed to reach significant concordance in the formal rating of the German-language websites.

Our study found no significant difference regarding the quality of content and formality between German- and English-language websites, as there are highly rated as well as poorly rated websites in both languages. While physicians might find high-quality international websites suitable for recommendation to patients familiar with the English language, consulting doctors and patients are also at risk of primarily finding websites of poor quality that should not be recommended regarding an evidence-based integrative treatment counseling.

Websites with poor quality of content or formality bear a high risk for the health and treatment success of cancer patients. Through poor formality there is the danger of misunderstanding and misinterpreting information. A lack of transparency leads to difficulties in estimating whether information is trustworthy and reliable or primarily the result of marketing and advertising. Through poor content the probability of harm for the patient is even higher. Multiple studies prove that misinformation influences people’s beliefs and actions to a great extent, regardless of the information’s validity [33, 34]. Recent studies by Spiteri Cornish et al. and Ruani et al. show that people frequently obey unscientific recommendations they read online. For example, this leads to the overestimation of functional foods in comparison to natural, unprocessed foods or the implementation of potentially dangerous behaviors like specific food group exclusion or overdosing on vitamins [35, 36]. Therefore, websites with poor content and misinformation bear a high risk for patients who search for advice online. By recommending only specific foods or food groups, or by limiting the amount of food intake, there is a real danger for patients to induce vitamin deficiencies, to consume foods that interfere with their cancer treatment, such as chemotherapy, or to ultimately induce cachexia, simply through not eating enough and not receiving the adequate amount of nutrients [14, 37, 38]. Altogether, those factors result in the decline of therapy response, an increased probability of therapeutic toxicity and a poorer prognosis in general [14, 39].

Currently, as the number of patients who consider cancer diets as a genuine treatment option is rising, there is an increase in the number of patients who suffer from the negative effects of these diets on their well-being and treatment success. In Germany, the prevalence of people having experience with CAM therapies in general, varies from 48% to 70% [18, 40], as they are seen as an additional treatment option by general practitioners, and are included into the daily practice [41]. As shown in a study by Yun et al., an increasing amount of information on nutrition and other integrative medicine modalities can be found on national cancer center websites, proving the growing demand for web-based information regarding these subjects amongst cancer patients [42]. The actual use of complementary and alternative treatment methods, like cancer diets, among German cancer patients varies, with studies indicating a prevalence of 30–77% [17, 43, 44]. Furthermore, studies revealed a non-disclosure rate of up to 80%, indicating that most patients do not communicate their use of CAM therapies to their attending physician and therefore do not attune their complementary and alternative therapies to the original cancer treatment [18, 45]. By only receiving basic information and insufficient or contradictory advice from their general practitioner, patients naturally feel the need to search the Internet for more specific advice with the difficulty of differentiating reliable information from low quality content [46, 47]. Consequently, doctors must ensure to actively ascertain the patient’s expectations and personal stance regarding the use of cancer diets to avoid a negative impact on their therapy response and treatment outcome.

Furthermore, our study reveals that the order of search results does not correlate with good quality of content and formality in either the German or English language. With the number one search result obtaining 31.7% of the users clicks, followed by a steep decline with ultimately only 0.78% of searchers clicking on a website from the second page [25, 26], it can be concluded that patients are not guaranteed to find only high-quality websites when searching for information on cancer diets on Google or Bing. Therefore, doctors need to know which websites can be recommended in particular and provide patients with online sources that support an evidence-based integrative treatment counseling.

However, the findings of our study are limited by certain factors. We used a rather restricted search term for both languages, tested only two search engines and only assessed the first 16 websites. Yet, as this also reflects the behavior of most users, our methodology provided representative data. We also did not conduct a validation study for the content and formal criteria of our evaluation tool, as these criteria were derived from national and international acknowledged tools. Furthermore, neither of the raters has the perspective of a patient on this subject nor is a native English speaker. Although both evaluators possess very advanced levels of English, this could possibly have had an impact on the assessment of language on the English-language websites. Lastly, as concordance among the two raters for the German-language websites failed to reach significance, though the overall interrater concordance and concordance for the English-language websites was good, the involvement of a third rater is recommended for further studies. Ultimately, the results of our study lead to the question how consulting doctors and patients can gain confidence in assessing the quality of information and in distinguishing high-quality websites from the ones lacking in formality and content. First, the information given on websites should be compared to the core statements of official guidelines that are developed and provided by experts. As a previous study by Barrett et al., assessing the web-based diet and nutrition information for cancer patients provided by English-speaking national cancer organizations concludes, most of these official websites offer high-quality general nutrition recommendations as well as extensive general healthy eating advice and may therefore be used for orientation and comparison [47].

For instance, the German S3-Guideline of the German Society for Nutritional Medicine specifically recommends the avoidance of cancer diets, as a possible cause for malnutrition and physical harm. Instead of advising special diets and differentiating foods patients should or should not eat, it focuses on the adjustment of nutrition to side effects of treatment, as well as the prevention of malnutrition and cachexia. Furthermore, it recommends a thorough screening of patients for malnutrition, as well as the consultation of doctors and nutritionists [14].

Altogether, a system of evaluation is necessary for patients and practitioners to recognize highly appraised and reviewed information, while at the same time, the visibility on the World Wide Web of websites containing evidence-based information needs to be increased.

In their study, Liebl et al. defined several steps, which decrease the risk of patients consuming poor quality websites online and thus of misinformation and health risks [24]. Those steps include raising the visibility of websites with evidence-based information through the adaptation of search key words, the inclusion of social media links or backlinks from trustworthy organizations. Furthermore, the information should be kept up to date, websites should be regularly reviewed and preferably be written by highly qualified authors. An additional offer for counselling by website providers could also be beneficial [24]. Another study recommends a quality control system through peer review to improve the visibility of evidence-based information, while at the same time avoiding the publishing of false or hazardous information [23, 48]. It furthermore emphasizes the importance of improving the search rank on Google through optimizing website texts or using campaigns, again resulting in higher visibility [23].

To conclude, doctors should take a more active role in educating their patients on the benefits and risks of cancer diets, precisely ask about the use of CAM, and ultimately know the websites with good quality of evidence-based information that were identified in our study and that can be recommended to their patients in terms of an evidence-based integrative treatment counseling. By following these steps, the risk of misinformation and harm to the patient can be minimized, resulting in a more successful and straightforward treatment of cancer.

## Data Availability

The datasets used and analyzed during the current study are available from the corresponding author on reasonable request.
